# Transition from Laparotomy to Laparoscopic Repair of Congenital Duodenal Obstruction in Neonates: Our Early Experience

**DOI:** 10.3389/fped.2017.00203

**Published:** 2017-09-22

**Authors:** Min Jeng Cho, Dae Yeon Kim, Seong Chul Kim, Jung Man Namgoong

**Affiliations:** ^1^Department of Surgery, Ulsan University Hospital, Ulsan, South Korea; ^2^Division of Pediatric Surgery, University of Ulsan College of Medicine, Asan Medical Center, Seoul, South Korea

**Keywords:** duodenal obstruction, duodenoduodenostomy, laparoscopy, neonate, laparotomy

## Abstract

**Background:**

The aim of this report was to review our early experience of the last 7 years with repairs of congenital duodenal obstruction (CDO) to determine the efficacy and outcomes of laparoscopic repairs compared to laparotomy.

**Methods:**

A retrospective review was conducted on all neonate (<30 days) with CDO between 2009 and 2015. Patients with duodenal atresia, stenosis, web, and annular pancreas were included. Patients with only malrotation or delayed presentation were excluded.

**Results:**

Twenty-six neonates underwent laparoscopy and 30 underwent traditional laparotomy. The operative time was longer in the laparoscopic group (*P* = 0.001), but time to initiation of feeds and time to full feeds were similar for the laparoscopic and open groups. There was no mortality, anastomosis leakage, or stenosis in the laparoscopic group. Six laparoscopic cases required conversion to an open procedure (23%). In the earlier cases, the open conversion rate was high, but it decreased over time (*P* = 0.003).

**Conclusion:**

Laparoscopic repair is safe and effective for repair of CDO in neonates. Despite operative time was slightly longer in the laparoscopic group, clinical outcomes remained similar to the open group. For pediatric surgeon with experience in laparoscopic techniques, laparoscopic duodenoduodenostomy is a sufficient available procedure.

## Introduction

Advancements in minimally invasive surgical techniques in neonates over the last 20 years have led to pediatric surgeons attempting laparoscopic repair of congenital duodenal obstruction (CDO). The first to report laparoscopic repair of a duodenal atresia was Bax et al. in 2001 (1). Since that case report, a few small series have been reported (2, 3). However, in the early experience, there was a high rate of anastomotic leaks (4). As technical skills developed, such as transabdominal stay suture, running suture, and intracorporeal knot tying, the ability to stabilize the anastomosis improved, and Spilde et al. reported a good result of the repair using the U-clip technique (5, 6). Recently, several studies showing short-term outcomes for open and laparoscopic approaches have been reported (7–12). The aims of this report is to review our early experience of the last 7 years to determine the efficacy and outcomes of laparoscopic repair of CDO compared to laparotomy.

## Materials and Methods

After Institutional Review Board approval, a retrospective review was performed on patients with CDO who presented to Asan Medical Center between 2009 and 2015. Patients with duodenal atresia, stenosis, duodenal web, and annular pancreas were included in this study, and only neonates of less than 30 days of age at the time of operation were enrolled. Patients with only malrotation or with a delayed presentation were excluded. The data collected included patient age and weight at surgery, associated anomalies, operative procedures performed, operative time, any intraoperative complications, and postoperative course. CDO was managed laparoscopically or *via* an open approach based on the surgeon’s choice. Cases that started laparoscopically and were converted to open procedures were included in the laparoscopic group.

Descriptive statistics are listed as median ± range. Binary frequency data were compared using the Fisher’s exact test, and continuous variables were compared using the Mann–Whitney *U* test. Changes over time in open conversion rate and operative time were analyzed using the chi-square test for trend and the Kruskal–Wallis test. *P* value <0.05 was regarded as statistically significant.

### Operative Technique

Open treatment was performed *via* a traditional transverse laparotomy. In cases with duodenal web, an excision of the web was performed, and for cases without duodenal web, duodenoduodenostomy was performed.

**Figure 1:**
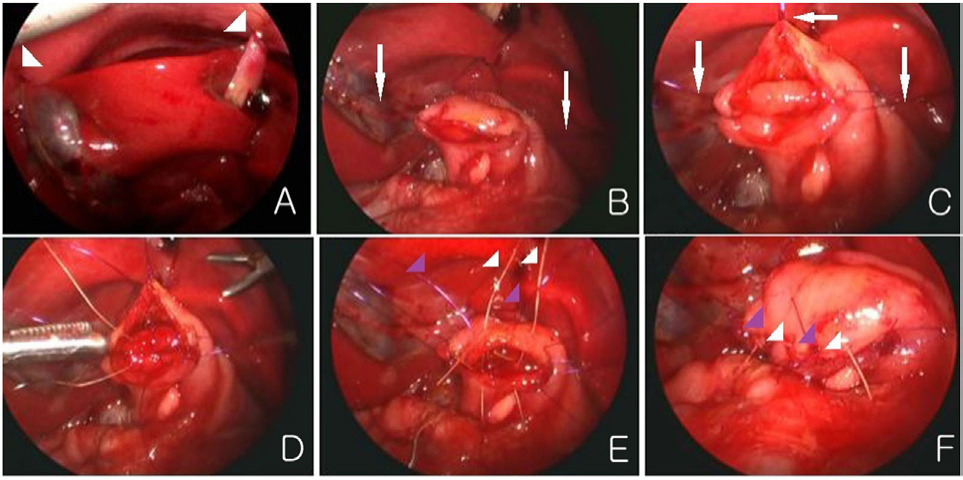
**(A)** Transabdominal sutures are placed through the falciform ligament and gall bladder (arrow head). **(B,C)** Two ends of the corners and the middle portion of proximal duodenum are led out through the skin for traction (white arrow). **(D–F)** The posterior and anterior anastomosis is made. Vicryl (white color) and PDS (purple color) are used alternately.

All laparoscopic procedures were performed using three trocars: one 4-mm port in the umbilicus and two 3-mm ports for the working instruments, one placed in the upper left quadrant and one in the right lower quadrant. A 4-mm 30° scope was used. A transabdominal stay sutures were placed through the falciform ligament and gallbladder for traction (Figure 1A). With the surgeon standing at the patient’s feet, the duodenum was Kocherized, and the dilated proximal and collapsed distal segments were identified. A proximal transverse and distal longitudinal duodenotomy was made, and then a diamond-shaped anastomosis was performed. After the corners were sutured, two ends of the corners and the middle portion of proximal duodenum were led out through the skin (at a convenient place) for traction (Figures 1B,C). We used interrupted sutures and intracorporeal knot tying. Vicryl was used for the corners of both sides, and Vicryl and PDS were used alternately for the rest of the sutures (Figures 1D–F). The distal bowel was examined in all cases to ensure that there was no obvious secondary atresia.

## Results

### Laparoscopic vs Laparotomy

For management of CDO, 26 neonates underwent laparoscopy, and 30 underwent traditional laparotomy. There was no difference in age or weight at the time of operation between the two groups (Table 1), but the open group patients had more associated anomalies (*P* = 0.01). Despite the longer operative time in the laparoscopic group (*P* = 0.001), the time to initiation of feeds and time to full feeds were similar between the two groups. The length of hospital stay in the open group was longer because several patients required further medical management for coexistent diseases. The follow-up period was shorter in the laparoscopic group than in the laparotomy group (*P* = 0.004).

**Table 1:** Baseline characteristics and outcomes.

	Laparoscopic (*n* = 26)	Open (*n* = 30)	*P* value
Birth weight (kg)	2.8 (1.8–3.9)	2.3 (0.9–3.6)	0.13
Gestational age (weeks)	37 (34–39)	35 (26–40)	0.16
Age at operation (days)	4 (2–9)	8 (1–29)	0.42
Weight at operation (kg)	2.5 (1.7–3.4)	2.2 (0.9–3.7)	0.82
Etiology of obstruction (*n*)			0.76
Duodenal atresia	17	16	
Duodenal web	8	8	
Annular pancreas	1	6	
Comorbidity (*n*)			0.01
Chromosome abnormality		4	
Congenital heart disease	2	6	
Esophageal atresia		1	
Imperforate anus	1	1	
Follow-up (months)	34.5 (16–86)	61.5 (17–98)	0.004
Operative time (min)	168 (119–250)	109 (50–166)	<0.001
Time to initiation of feeds (days)	8 (3–18)	10 (5–35)	0.33
Time to full feeds (days)	15 (7–26)	20 (8–94)	0.09
Overall length of stay (days)	17 (8–28)	20 (9–228)	0.008
Complication (*n*)	0	3	0.62
Mortality (*n*)	0	1	0.54

The laparoscopic group had no complications, while three patients in the open group experienced complications. In the open group, bowel obstruction due to postoperative adhesion occurred in two patients on the 15th and 50th day, and adhesiolysis, small bowel resection, and anastomosis were performed. One patient in the open group underwent R-Y duodenojejunostomy 10 months after the initial operation, due to stricture of the anastomosis. One patient in the open group who had coarctation died of cardiac issues.

### Laparoscopic Repair of CDO

**Figure 2:**
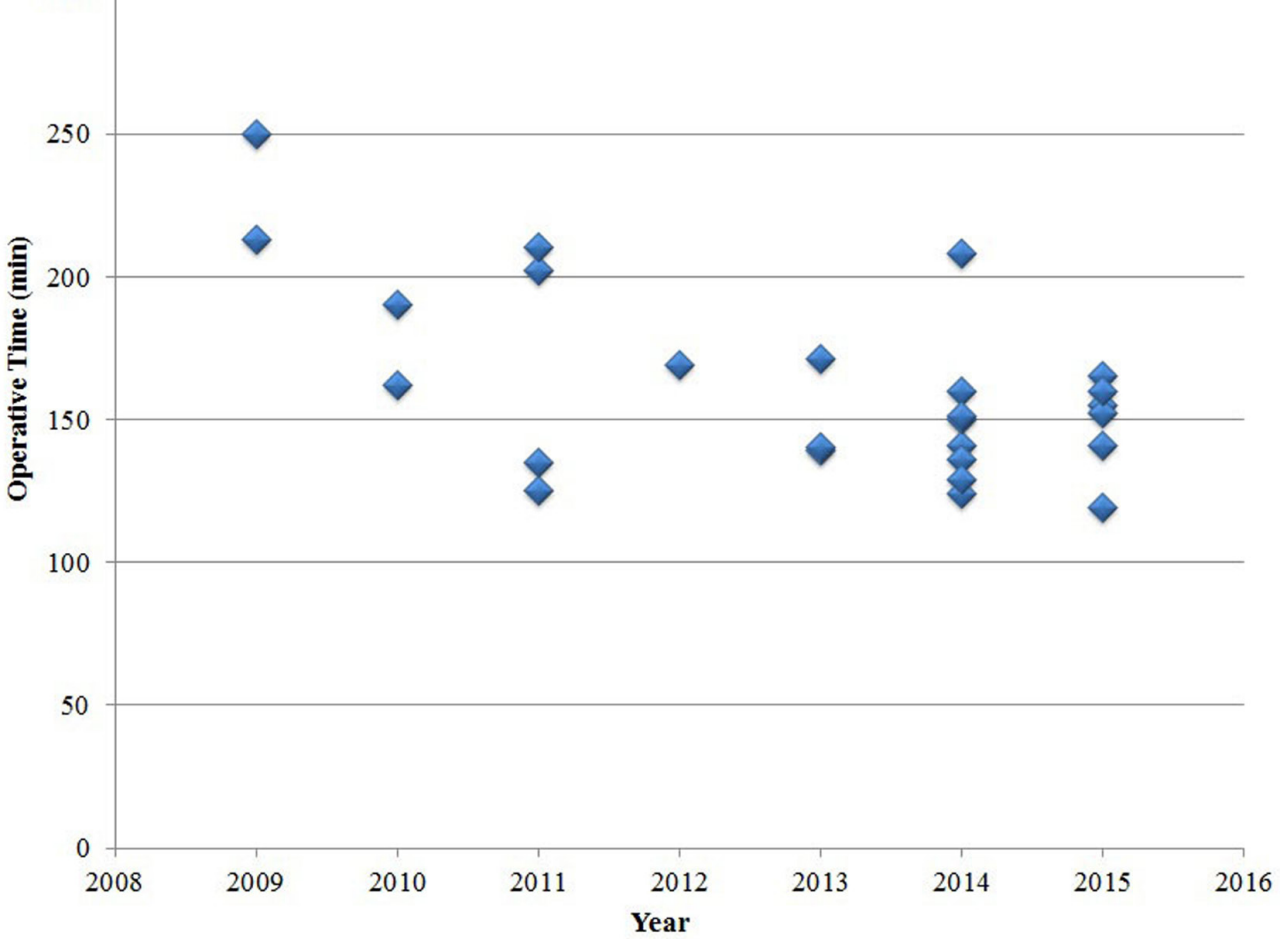
Operation time in the laparoscopic group.

**Figure 3:**
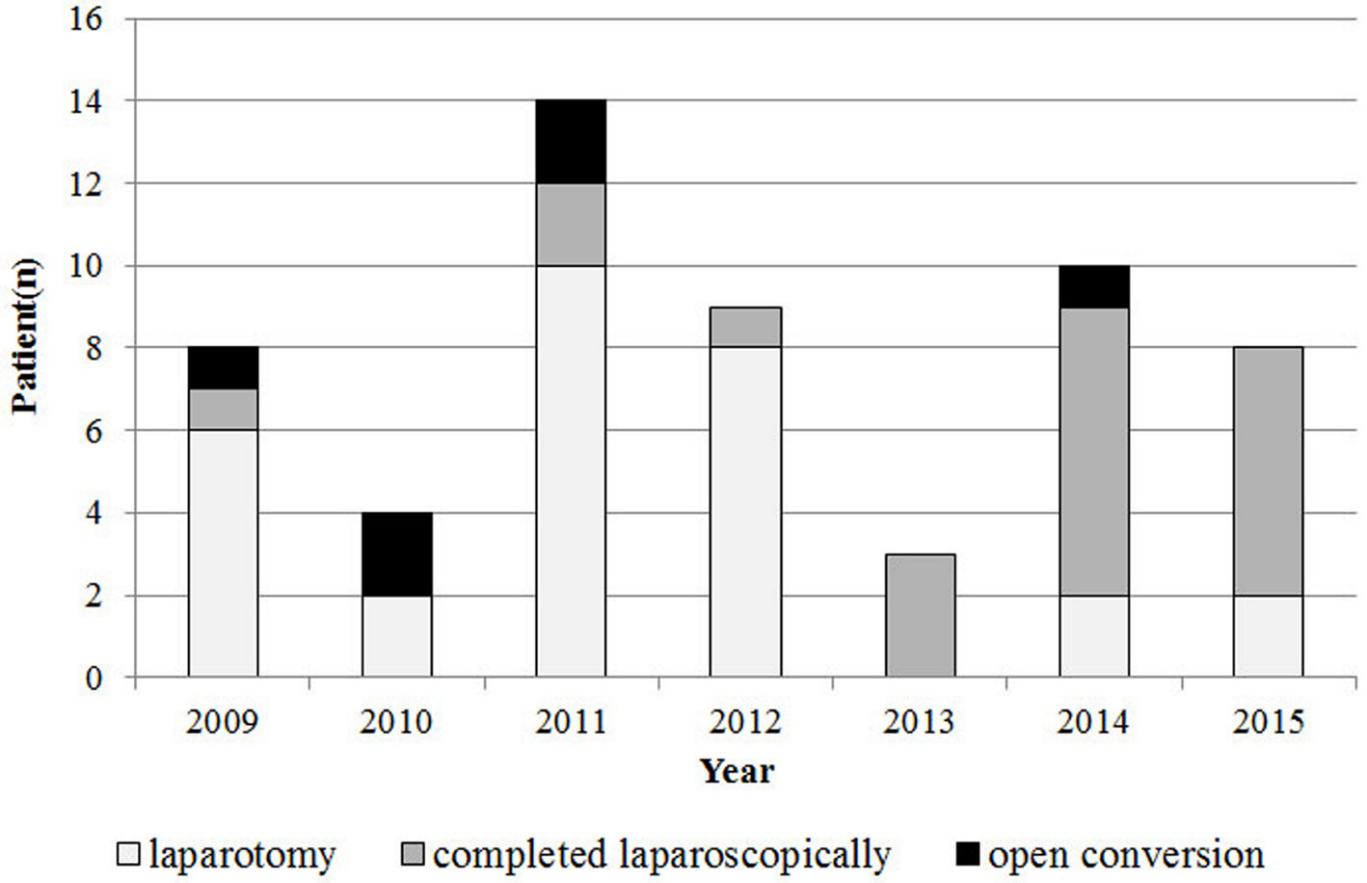
Laparoscopic and open repair by year.

Six laparoscopic cases required conversion to an open procedure (23%), most commonly due to poor visualization of the duodenum (*n* = 3). Other indications for conversion included bleeding (*n* = 2), and concern of perforation due to serosal injury (*n* = 1). There were no significant differences in age or weight at the time of the surgery between the 20 patients whose surgeries were completed laparoscopically and the 6 patients whose surgeries were converted to laparotomy. In the early experience stage, the conversion rate was high, but it decreased over time (*P* = 0.003). The operative time for laparoscopic repair decreased over time, but this decrease was not statistically significant (*P* = 0.15) (Figure 2). The relative frequency of laparoscopic repair increased over time (Figure 3), and the rate of laparoscopic repair has been higher than that of the open procedure since 2013.

## Discussion

The main concerns of performing laparoscopic repair of CDO in neonates are anastomotic stricture and leakage, and another concern is not being able to identify distal patency. A bulbous duodenum makes the formation of the anastomosis more difficult especially in laparoscopic approach. Our traction method was performed on the corners of both sides of the anastomosis using the abdominal wall, and this method not only provides good visualization but also helps the anastomosis be formed evenly and safely. There are some studies that mentioned similar traction techniques (4, 13), but our method has the advantage of not requiring an additional trocar, and it allows two working instruments to be used freely. In addition, we believe that the interrupted suture was more effective at preventing stricture than the running suture. By using Vicryl and PDS alternately, the pros and cons of the two suture materials complemented each other; furthermore, the spacing between sutures was even because the different colors of the sutures allowed them to be easily distinguished at the anastomosis. In other studies, running sutures was frequently used to prevent leakage (7, 8, 14). We used interrupted sutures with adjusted by traction, and no cases developed leakage. With regard to distal patency, the incidence of secondary atresia was extremely low (2%), it is sufficient to carefully inspect the intestine during the laparoscopic procedure. Although an internal web is difficult to identify, it is similarly difficult to identify with the open approach.

In our study, the median time to initiation of feeds, achievement of full feeds, and length of hospitalization in the laparoscopic group were similar to those of most other laparoscopic series (7–10, 15). Another study showed that the laparoscopic approach reduces the length of hospitalization and time to initial feeds (5); however, it is thought that the difference between the feeding protocols based on the upper gastrointestinal contrast studies. The long-term results in our limited series of laparoscopic procedures were similar to those obtained with the open technique. Although it was hard to confirm the statistical significance of differences in complications between the two groups because of the small number of cases, duodenal obstruction due to adhesion occurred in two patients in the open group after the operation. One of the biggest benefits of the laparoscopic approach, in addition to the cosmetic aspect, is the reduction of adhesion due to less touch. The reduction of adhesion and duodenal obstruction with the laparoscopic approach is a particularly significant advantage when treating pediatric patients, when the laparoscopic approach results in similar outcomes to the open approach.

In this study, the open conversion rate significantly decreased over time (*P* = 0.003). The operative time of laparoscopic repair decreased over time, but this decrease was not statistically significant. The operative time in our laparoscopic series appeared to be longer than in other reported contemporary series (7–9, 14). One reason for this finding may be that we performed interrupted sutures instead of running sutures and we expect that the operative time will be decreased as experience is gained.

This study is limited by its retrospective nature and susceptibility to selection bias. We attempted to minimize the selection bias by excluding patients >30 days of age. The small intraabdominal working space in neonates makes laparoscopic operations more difficult and time consuming than in older children. Moreover, patients only with malrotation were excluded because the Ladd operation was conducted on them, which does not require the formation of an anastomosis and, therefore, could not allow for a proper comparison. Nonetheless, this study could have had selection bias, as patients with comorbidities, such as a heart anomaly, were more frequently treated using open approach; comorbidities did not affect postoperative complications in any cases, but the length of hospitalization and postoperative ventilator days could not be compared. In the open group, for patients with web, it was excised, but duodenoduodenostomy was performed on all patients in the laparoscopic group. Studies have reported good results with laparoscopic excision of web (7, 13, 14), but we prefer laparoscopic duodenoduodenostomy than duodenotomy with web excision because of the possibility of injury to the ampulla in small neonates with the latter procedure. Finally, the laparoscopic group has a significantly shorter follow-up period. Late complications in the laparoscopic group are difficult to assess completely, and there are limitations that cannot accurately compare the complications of the two groups.

## Conclusion

This study demonstrates laparoscopic repair is a safe alternative to traditional open repair of CDO. For pediatric surgeon with experience in laparoscopic techniques, laparoscopic duodenoduodenostomy is a sufficient available procedure.

## Ethics Statement

This study was approved by Asan Medical Institutional Review Board (AMC IRB 2017-1698).

## Author Contributions

All the authors contributed to the design and interpretation of the study. MC and DK proposed the study and wrote the first draft. SK and JN collected the data.

## Conflict of Interest Statement

MC, DK, SK, and JN have no conflicts of interest or financial ties to disclose.

## References

[1] Bax NM Ure BM van der Zee DC van Tuijl I. Laparoscopic duodenoduodenostomy for duodenal atresia. Surg Endosc (2001) 15(2):217. 12200660 10.1007/BF03036283

[2] Rothenberg SS. Laparoscopic duodenoduodenostomy for duodenal obstruction in infants and children. J Pediatr Surg (2002) 37(7):1088–9. 10.1053/jpsu.2002.33882 12077777

[3] Frantzides CT Madan AK Gupta PK Smith C Keshavarzian A. Laparoscopic repair of congenital duodenal obstruction. J Laparoendosc Adv Surg Tech A (2006) 16(1):48–50. 10.1089/lap.2006.16.48 16494548

[4] van der Zee DC. Laparoscopic repair of duodenal atresia: revisited. World J Surg (2011) 35(8):1781–4. 10.1007/s00268-011-1147-y 21604051 PMC3127017

[5] Spilde TL St Peter SD Keckler SJ Holcomb GW III Snyder CL Ostlie DJ. Open vs laparoscopic repair of congenital duodenal obstructions: a concurrent series. J Pediatr Surg (2008) 43(6):1002–5. 10.1016/j.jpedsurg.2008.02.021 18558173

[6] Valusek PA Spilde TL Tsao K St Peter SD Holcomb GW III Ostlie DJ. Laparoscopic duodenal atresia repair using surgical U-clips: a novel technique. Surg Endosc (2007) 21(6):1023–4. 10.1007/s00464-007-9211-2 17623253

[7] Jensen AR Short SS Anselmo DM Torres MB Frykman PK Shin CE Laparoscopic versus open treatment of congenital duodenal obstruction: multicenter short-term outcomes analysis. J Laparoendosc Adv Surg Tech A (2013) 23(10):876–80. 10.1089/lap.2013.0140 24079961 PMC3967418

[8] Hill S Koontz CS Langness SM Wulkan ML. Laparoscopic versus open repair of congenital duodenal obstruction in infants. J Laparoendosc Adv Surg Tech A (2011) 21(10):961–3. 10.1089/lap.2011.0069 22129146

[9] Chung PH Wong CW Ip DK Tam PK Wong KK. Is laparoscopic surgery better than open surgery for the repair of congenital duodenal obstruction? A review of the current evidences. J Pediatr Surg (2017) 52(3):498–503. 10.1016/j.jpedsurg.2016.08.010 27622585

[10] Mentessidou A Saxena AK. Laparoscopic repair of duodenal atresia: systematic review and meta-analysis. World J Surg (2017) 41(8):2178–84. 10.1007/s00268-017-3937-3 28258456

[11] Chiarenza SF Bucci V Conighi ML Zolpi E Costa L Fasoli L Duodenal atresia: open versus MIS repair-analysis of our experience over the last 12 years. Biomed Res Int (2017) 2017:4585360. 10.1155/2017/4585360 28326320 PMC5343219

[12] Parmentier B Peycelon M Muller CO El Ghoneimi A Bonnard A. Laparoscopic management of congenital duodenal atresia or stenosis: a single-center early experience. J Pediatr Surg (2015) 50(11):1833–6. 10.1016/j.jpedsurg.2015.05.007 26093906

[13] Burgmeier C Schier F. The role of laparoscopy in the treatment of duodenal obstruction in term and preterm infants. Pediatr Surg Int (2012) 28(10):997–1000. 10.1007/s00383-012-3136-0 22991205

[14] Li B Chen WB Zhou WY. Laparoscopic methods in the treatment of congenital duodenal obstruction for neonates. J Laparoendosc Adv Surg Tech A (2013) 23(10):881–4. 10.1089/lap.2013.0097 23968252

[15] Kay S Yoder S Rothenberg S Laparoscopic duodenoduodenostomy in the neonate. J Pediatr Surg (2009) 44(5):906–8. 10.1016/j.jpedsurg.2009.01.025 19433167

